# Highly Stretchable, Adhesive, Biocompatible, and Antibacterial Hydrogel Dressings for Wound Healing

**DOI:** 10.1002/advs.202003627

**Published:** 2021-03-05

**Authors:** Zifeng Yang, Rongkang Huang, Bingna Zheng, Wentai Guo, Chuangkun Li, Wenyi He, Yingqi Wei, Yang Du, Huaiming Wang, Dingcai Wu, Hui Wang

**Affiliations:** ^1^ Department of Colorectal Surgery The Sixth Affiliated Hospital, Sun Yat‐sen University, Guangdong Institute of Gastroenterology Guangdong Provincial Key Laboratory of Colorectal and Pelvic Floor Diseases Guangzhou 510655 P. R. China; ^2^ PCFM Lab and GDHPRC Lab School of Chemistry Sun Yat‐sen University Guangzhou 510275 P. R. China

**Keywords:** adhesive, antibacterial dressings, hydrogel dressings, stretchable materials, wound healing

## Abstract

Treatment of wounds in special areas is challenging due to inevitable movements and difficult fixation. Common cotton gauze suffers from incomplete joint surface coverage, confinement of joint movement, lack of antibacterial function, and frequent replacements. Hydrogels have been considered as good candidates for wound dressing because of their good flexibility and biocompatibility. Nevertheless, the adhesive, mechanical, and antibacterial properties of conventional hydrogels are not satisfactory. Herein, cationic polyelectrolyte brushes grafted from bacterial cellulose (BC) nanofibers are introduced into polydopamine/polyacrylamide hydrogels. The 1D polymer brushes have rigid BC backbones to enhance mechanical property of hydrogels, realizing high tensile strength (21–51 kPa), large tensile strain (899–1047%), and ideal compressive property. Positively charged quaternary ammonium groups of tethered polymer brushes provide long‐lasting antibacterial property to hydrogels and promote crawling and proliferation of negatively charged epidermis cells. Moreover, the hydrogels are rich in catechol groups and capable of adhering to various surfaces, meeting adhesive demand of large movement for special areas. With the above merits, the hydrogels demonstrate less inflammatory response and faster healing speed for in vivo wound healing on rats. Therefore, the multifunctional hydrogels show stable covering, little displacement, long‐lasting antibacteria, and fast wound healing, demonstrating promise in wound dressing.

## Graphical abstract

A new class of highly stretchable, adhesive, biocompatible, and antibacterial hydrogel dressing is designed and prepared by synergizing biocompatible polyacrylamide hydrogel scaffold, biological adhesive polydopamine, and antibacterial polymer brush grafted from bacterial cellulose nanofibers.

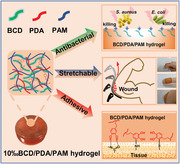

## 1 Introduction

Wound dressing has been an important branch of biomedical materials research.^[^ ^1^ ^]^ Skin damage caused by abrasion after falling and clinical incision after surgery are the most common wounds in real life.^[^ ^2^ ^]^ Compared with the wounds at flat areas of human body, it is still challenging to treat the wounds at special areas such as joints, popliteal fossae, axillae, and muscle folds. Poor adhesive performance, difficult fixation, and incomplete coverage are the main reasons. Moreover, in clinic, most wounds are sterilized with 75% alcohol or iodine, followed by covering with cotton gauzes. Therefore, regular disinfection and dressing replacement are necessary due to lack of antibacterial property of cotton gauze. In addition, fixed cotton gauze dressing needs to be taped, and sometimes, the skin is allergic to tape material. To this end, designing a stretchable, adhesive, antibacterial, and biocompatible dressing is of great clinical significance.

Hydrogels are a class of 3D network gels formed by chemical and/or physical crosslinking.^[^ ^3^ ^]^ Because of their superior biocompatibility, controllable physical properties, natural drug‐loading structure, and abundant functional groups, hydrogels have gradually become a hotspot of medical wound dressing. To date, hydrogels have been successfully applied to treat skin defects,^[^ ^4^ ^]^ infected wounds,^[^ ^5^ ^]^ burn wounds,^[^ ^6^ ^]^ diabetic feet,^[^ ^7^ ^]^ and wet wounds inside the body.^[^ ^8^ ^]^ Based on the healing requirements of different wounds, one or more functions such as good tissue adhesion, excellent mechanical property, antibacterial capability, cell crawling promotion, physical contraction, local immune regulation, and antitumor property have been implanted in the hydrogel dressings, such as Cur‐QCS/PF,^[^ ^5^ ^]^ QCSP/PEGS‐FA^[^ ^9^ ^]^ , OSA‐DA‐PAM,^[^ ^3^ ^]^ PDA@AgNPs/CPHs,^[^ ^10^ ^]^ NPs‐P‐PAA,^[^ ^11^ ^]^ and STP^[^ ^12^ ^]^ hydrogels. However, the existing hydrogel dressings were difficult to achieve a satisfactory balance among the multiple functions. For example, due to the presence of quaternary ammonium salt, both Cur‐QCS/PF^[^ ^5^ ^]^ and QCSP/PEGS‐FA^[^ ^9^ ^]^ hydrogels showed excellent antibacterial property, but their tensile strain (below 100%) and adhesion strength (less than 8 kPa) were not satisfactory. OSA‐DA‐PAM^[^ ^3^ ^]^ and STP^[^ ^12^ ^]^ hydrogel dressings had good adhesion and stretching performances, but no antibacterial property. With the catechol structure and nanosilver, PDA@AgNPs/CPHs^[^ ^10^ ^]^ and NPs‐P‐PAA^[^ ^11^ ^]^ hydrogels exhibited excellent tissue adhesion and antibacterial properties, but use of silver‐functionalized biomedical materials could give rise to cumulative toxic effect of heavy metal in organisms.^[^ ^13^ ^]^ Therefore, it is still highly challenging in preparing a hydrogel dressing with ideal tissue adhesion, good stretchability, broad‐band antibacterial capability, cell crawling promotion, and bio‐compatibility by a facile and efficient material design.

Herein, a new class of highly stretchable, adhesive, biocompatible, and antibacterial hydrogel dressings is designed and prepared by introducing poly(diallyl dimethyl ammonium chloride) (pDADMAC) brushes grafted from bacterial cellulose (BC) nanofibers (BC‐*g*‐pDADMAC, BCD) into polydopamine/polyacrylamide (PDA/PAM) hydrogels. For the as‐prepared multifunctional BCD/PDA/PAM hydrogels, the PAM component has good biocompatibility and stable crosslinking structure, and is thus used as the hydrogel scaffold.^[^ ^3^ ^]^ Inspired by the biological adhesion of dopamine from mussel,^[^ ^14^ ^]^ the PDA component has abundant catechol groups and thus can adhere to various surfaces, especially special areas needing large movements. More importantly, the BCD component not only has rigid BC backbones to enhance the mechanical property of hydrogels, but also has pDADMAC brushes with broad spectrum and low toxic positively charged quaternary ammonium groups for providing high‐efficiency and long‐lasting antibacterial performance. Moreover, the positively charged pDADMAC brushes could help attract negatively charged epidermis cells and thus promote cell crawling and proliferation.^[^ ^15^ ^]^ Therefore, our BCD/PDA/PAM hydrogels have superior mechanical behaviors with high tensile strength (21–51 kPa), large tensile strain (899–1047%) and ideal compressive performance, and demonstrate stable covering, little displacement, long‐lasting antibacteria and fast wound healing.

## 2 Results and Discussion

### 2.1 Structure Characterizations

**Figure 1:**
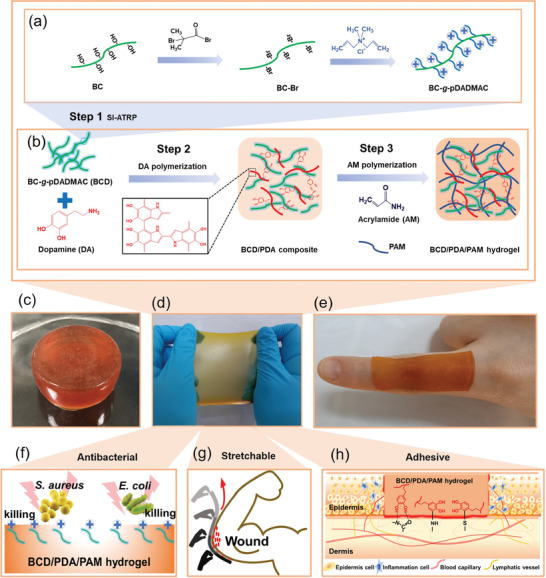
Schematic of the stretchable, adhesive, and antibacterial hydrogel. a) Preparation of BCD by using SI‐ATRP to graft pDADMAC from BC. b) Formation of BCD/PDA/PAM hydrogel. c–e) Different shapes of 10‰BCD/PDA/PAM hydrogel. f) Quaternary ammonium salts of BCD provide the antibacterial property to BCD/PDA/PAM hydrogel. g) Stretchable BCD/PDA/PAM hydrogel covers the elbow joint completely. h) Catechol groups of PDA component promote tissue adhesion of hydrogel.

**Figure 2:**
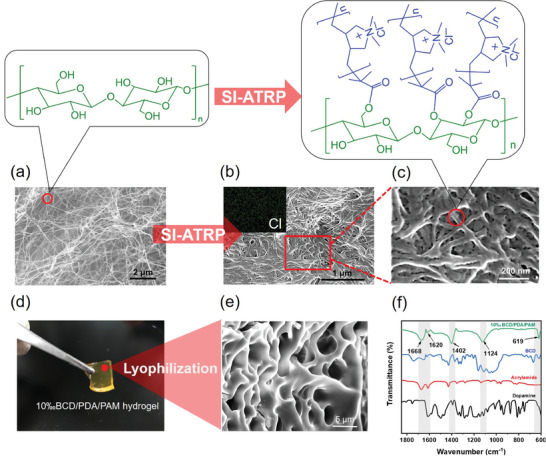
SEM images of a) BC and b,c) BCD; the inset of (b) is the element mapping of Cl. d) Digital photo and e) SEM image of 10‰BCD/PDA/PAM hydrogel. f) FT‐IR spectra of dopamine, acrylamide, BCD and 10‰BCD/PDA/PAM hydrogel.

BC has a natural nanofiber network structure with good hydrophilicity, mechanical property, and biocompatibility, which has a broad application prospect in biomedical dressings.^[^ ^16^ ^]^ Surface‐initiated atom transfer radical polymerization (SI‐ATRP) is a highly efficient active/controllable polymerization system and can be used in surface functionalization of BC with abundant hydroxyl groups.^[^ ^17^ ^]^ pDADMAC chains were grafted from nanofibers of BC via SI‐ATRP, leading to formation of BCD (**Figure** **1**a).The original smooth and fine nanofibers of BC became rough after grafting pDADMAC (**Figure** **2**a–c). Element mapping showed the presence of chlorine on the surface of BCD, confirming the successful polymeric modification of BC (Figure 2b, inset). The X‐ray photoelectron spectroscopy (XPS) spectrum of BC showed O_1s_ (533.1 eV) and C_1s_ (287.1 eV) peaks (Figure S1a, Supporting Information); peaks at 287.9, 286.3, and 284.7 eV of the deconvoluted high resolution C_1s_ spectrum were mainly attributed to bonds of O—C—O, C—O, and C—C/C—H of BC, respectively (Figure S1b, Supporting Information).^[^ ^17^, ^18^ ^]^ After grafting pDADMAC, new peaks appeared at 197.1, 268.0, and 402.1 eV, which were assigned to Cl_2p_, Cl_2s_, and N_1s_, respectively (Figure S1c, Supporting Information); peak at 286.2 eV was mainly attributed to C—N/C—O bonds of BCD (Figure S1d, Supporting Information).^[^ ^19^ ^]^ Fourier transform infrared (FT‐IR) was also used to study the structure evolution of BC, initiator‐functionalized BC (BC‐Br) and BCD (Figure S2, Supporting Information). Compared to BC, a new peak appeared at 1735 cm^−1^ for BC‐Br, which was attributed to carbonyl (C=O) stretching vibration;^[^ ^20^ ^]^ new peaks at 848 and 1479 cm^−1^ for BCD were ascribed to the characteristic bands of C—N and methyl (—CH_3_) groups of quaternized ammonium,^[^ ^17^, ^21^ ^]^ indicating pDADMAC was successfully grafted.

The preparation of BCD/PDA/PAM hydrogels was achieved by incorporating BCD into PDA/PAM hydrogel network. For the traditional PDA/PAM hydrogel, prepolymerization of dopamine was usually carried out in a weak alkaline (pH = 8) environment,^[^ ^22^ ^]^ which could affect the stability of acidic reactants. Here, BCD was firstly dispersed in the aqueous dopamine solution. Considering dopamine could prepolymerize under the action of oxidants,^[^ ^23^ ^]^ ammonium persulfate (APS) was used for prepolymerization of dopamine in our study (Figure 1b, Step 2), which can avoid use of conventional alkaline condition and ice bath. It took 25 min for the color of the solution to gradually change from white to brown in room temperature with a high‐speed stirring (Figure S3, Supporting Information). Subsequently, acrylamide and crosslinking agent were added into the reaction system (Figure 1b, Step 3). The remaining APS was directly used as initiator for synthesis of BCD/PDA/PAM hydrogels (e.g., 10‰BCD/PDA/PAM hydrogel, Figure 1c). With a reaction in a 60 °C chamber for 3 h, the gelation was completed (Figure S4, Supporting Information). Compared with other studies,^[^ ^14a^ ^]^ the whole process of our hydrogel preparation was simple and mild, which was free of pH control and ice bath. The as‐obtained 10‰BCD/PDA/PAM hydrogel showed stretchable and adhesive properties (Figure 1d,e,g). The rich catechol groups from PDA component of the hydrogel product could enhance cell affinity, tissue adhesion, and cell proliferation (Figure 1h, S5a, Supporting Information), which has been applied to many biomedical hydrogels.^[^ ^24^ ^]^ Addition of BCD component could improve the mechanical strength and cell affinity of hydrogel.^[^ ^16^, ^25^ ^]^ BCD is a kind of cationic polyelectrolyte brush and can form porous networks by the interconnection of nanofibers, which is conducive to cell adhesion and crawling. Meanwhile, the positively charged quaternary ammonium salts of BCD facilitate attraction of negatively charged normal cells by electrostatic interaction;^[^ ^26^ ^]^ the increase of surface charge for biomaterials could enhance the number of biologically available surface‐attached proteins, leading to an increased cell spreading area after adhesion.^[^ ^27^ ^]^ Therefore, the incorporation of PDA and BCD components can enhance the cell affinity of 10‰BCD/PDA/PAM hydrogel (Figure S5b, Supporting Information). Due to presence of positively charges, the zeta potential of BCD/PDA/PAM hydrogel was measured to be 1.42–3.78 mV (pH = 6.5) or 1.64–3.35 mV (pH = 7.2), and became higher with increasing the BCD content (Figure S6, Supporting Information). The positive charges would endow the hydrogel with antibacterial performance (Figure 1f) and promotion of cell crawling and proliferation (Figure S5, Supporting Information).^[^ ^26^, ^28^ ^]^

10‰BCD/PDA/PAM hydrogel was transparent light brown (Figure 2d), which was different from transparent and colorless BC/PAM hydrogel (Figure S7a, inset, Supporting Information) and brown PDA/PAM hydrogel (Figure S7b, inset, Supporting Information). After lyophilization, 10‰BCD/PDA/PAM hydrogel showed a porous network with a pore diameter of 3–5 µm (Figure 2e). For BC/PAM and PDA/PAM hydrogels, their pore diameters were ≈5–20 µm (Figure S7, Supporting Information). As shown in FT‐IR spectrum of 10‰BCD/PDA/PAM hydrogel (Figure 2f), the peak at 1668 cm^−1^ was ascribed to C=O stretching of PAM; the peak at 1620 cm^−1^ was the N—H deformation peak for primary amine deriving from PDA, demonstrating the successful polymerization of dopamine in the system; the C—N stretching peak at 1402 cm^−1^ was from primary amide of both PDA and PAM in the hydrogel; the —NH_2_ in‐plane rocking peak at 1124 cm^−1^ mainly derived from PAM; the characteristic peak of BC at 619 cm^−1^ in the fingerprint region proved the existence of BCD.^[^ ^22e^ ^]^

### 2.2 Mechanical and Adhesive Properties

The network of BCD/PDA/PAM hydrogels was maintained by both noncovalent bonding (e.g., hydrogen bonding, van der Waals force, and electrostatic interaction) and covalent bonding of crosslinked polymers. Due to the existence of noncovalent bonds, 10‰BCD/PDA/PAM hydrogel showed a valuable self‐healing performance (Figure S8, Supporting Information).^[^ ^22^, ^29^ ^]^ The cut 10‰BCD/PDA/PAM hydrogel was healed within 30 min and could be stretched without cracking after 2 h healing (Figure S8a,b, Supporting Information). Meanwhile, the sample with 30 min healing showed a good healing effect and no re‐fracture was observed when attached to the left index finger with the movement of joints (Figure S8c, Supporting Information). As a result, the self‐healed hydrogel would be enough to meet the requirements of dressing.

**Figure 3:**
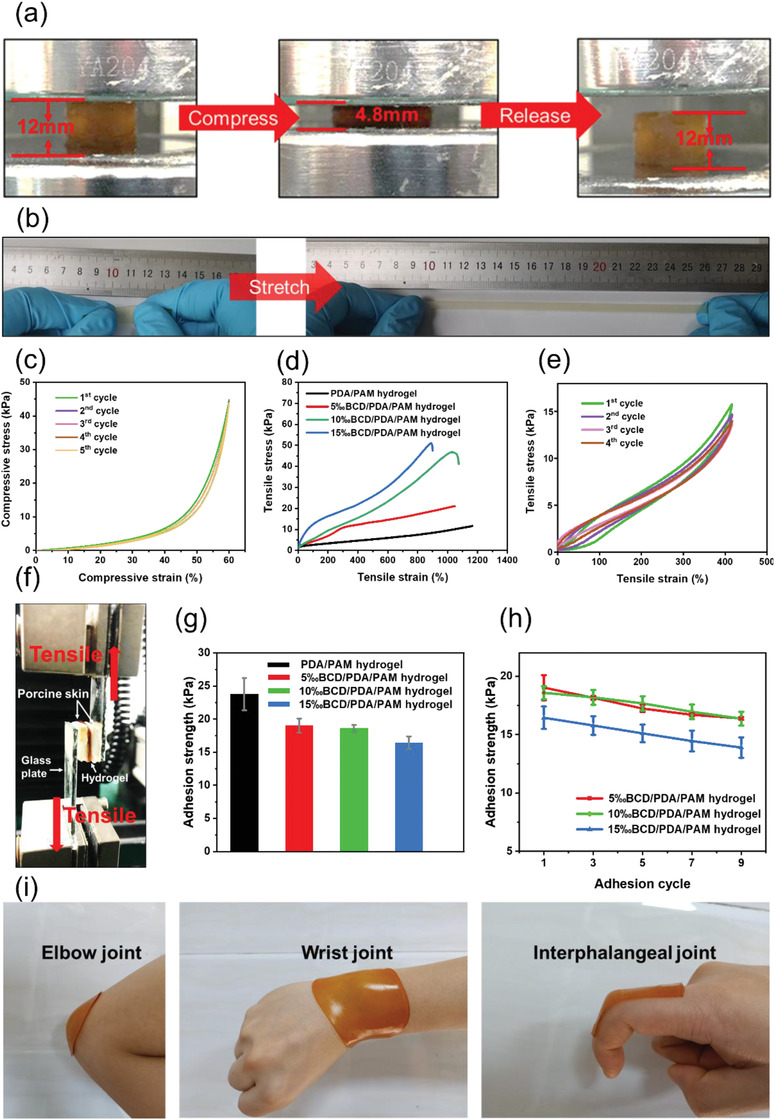
a) 10‰BCD/PDA/PAM hydrogel was compressed up to the strain of 60% and recovered to its original shape after it was released. b) Digital photos of the tensile test of 10‰BCD/PDA/PAM hydrogel, showing its high stretchability. c) Cyclic compressive loading–unloading test of 10‰BCD/PDA/PAM hydrogel. d) Tensile stress–strain curves of BCD/PDA/PAM hydrogels with different BCD contents and PDA/PAM hydrogel. e) Cyclic tensile test of 10‰BCD/PDA/PAM hydrogel under a tension of 400%. f) Digital photo of lap shear test using porcine skin. g) Adhesion strengths of BCD/PDA/PAM and PDA/PAM hydrogels. The error bars showed standard deviation (*n* = 3). h) Changes of adhesion strength with different adhesion cycles for BCD/PDA/PAM hydrogels. The error bars showed standard deviation (*n* = 3). i) Digital photos of 10‰BCD/PDA/PAM hydrogel adhered to the body's frequently moving joints.

With a network system of physical and chemical crosslinking, BCD/PDA/PAM hydrogels have good elasticity and toughness. Under compressive loading, 10‰BCD/PDA/PAM hydrogel could withstand 60% of compressive strain under a stress of 45 kPa and recover to its original shape after removal of the load (**Figure** **3**a; Figure S9, Supporting Information). After 5 cycles of loading‐unloading test, the loops were similar to that of the first cycle (Figure 3c). Therefore, the hydrogel had stable elasticity and toughness, compared to the reported pure PAM hydrogel.^[^ ^22a^ ^]^ The good resilience to withstand large deformation and high compressive strength made it qualified to meet the demand of toughness as hydrogel dressing.

As shown in Figure 3b, 10‰BCD/PDA/PAM hydrogel could be stretched to about 10 times of its initial length. Tensile strains of BCD/PDA/PAM hydrogels with different BCD ratios ranged from 899% to 1047% (Figure 3d), better than the other hydrogel wound dressing (normally lower than 100%).^[^ ^5^, ^24^, ^30^ ^]^ Addition of BCD enhanced the degree of crosslinking, which slightly decreased the strain compared with PDA/PAM hydrogel (1207%), but improved the toughness of hydrogel. The tensile strength of BCD/PDA/PAM hydrogels increased with an increase of BCD content, indicating that BCD had a reinforcing effect. The fracture tensile strength greatly increased from 21 to 51 kPa as an increase of the BCD ratio from 5‰ to 15‰, compared with PDA/PAM hydrogel (11 kPa). During cyclic tensile tests, a small degree of stress reduction was observed after the first cycle, which was attributed to the toughness of BCD network (Figure 3e). In the later cycles, the stress kept almost the same value, indicating a small energy dissipation.

BCD/PDA/PAM hydrogels showed excellent adhesion to various substrates. For example, 10‰BCD/PDA/PAM hydrogel could be directly adhered to the surfaces of plastic, glass, porcine skin, and human skin with different bearing weights (Figure S10a, Supporting Information). The tissue adhesive mechanism of our hydrogels was attributed to catechol groups of PDA component that had high binding affinity to amines, thiols, and imidazoles in peptides and proteins of tissue (Figure 1h).^[^ ^24^, ^31^ ^]^ As 10‰BCD/PDA/PAM hydrogel was peeled from the porcine and human skins, visible sticky fibrils (indicated by red arrows) formed at the hydrogel‐skin interfaces, indicating strong bonding (Figure S10b, Supporting Information).^[^ ^29^ ^]^ Although sticky fibrils formed during the separation, no residual hydrogel remained on the skin and no allergy was observed after a 24 h adhesion (Figure S10c, Supporting Information), indicating that 10‰BCD/PDA/PAM hydrogel had good biocompatibility.^[^ ^24c^ ^]^ To evaluate the potential utilization of BCD/PDA/PAM hydrogels as medical adhesives, in vitro lap shear testing was performed using porcine skin (Figure 3f).^[^ ^8^, ^32^ ^]^ The adhesion strength was maintained between 15 and 20 kPa (Figure 3g), higher than the commercial fibrin glue and other hydrogel dressings (normally 7.3–15.38 kPa, Figure S11, Supporting Information).^[^ ^5^, ^12^, ^29^ ^]^ The bonding of BCD and PDA in BCD/PDA/PAM hydrogels might consume a small part of hydroxyl groups of catechol groups. In addition, introduction of BCD improved the stiffness of BCD/PDA/PAM hydrogels, which would affect the polymer flexibility and then reduce the co‐adhesion of the interface. These might be the reasons for the reduction of adhesion strength of BCD/PDA/PAM hydrogels as compared with PDA/PAM hydrogel (Figure 3g).^[^ ^14^, ^31^ ^]^ To further verify the repeatability of adhesion, 9‐cycle peeling‐off testing was performed on porcine skin (Figure 3h). The adhesion strength of BCD/PDA/PAM hydrogels decreased slightly with an increase of cycling numbers, but still could meet the adhesive requirements for hydrogel dressing. Furthermore, in order to test the stability of hydrogels by repeated stretching, 10‰BCD/PDA/PAM hydrogel was fixed on the dynamic skin surfaces, including elbow, wrist, and interphalangeal joints. It was found that the position of the hydrogel was fixed without any retraction or rupture during this test process (Figure 3i; Figure S12, Supporting Information). These above good mechanical properties and excellent adhesion performances allowed BCD/PDA/PAM hydrogels to meet the demands of repeating stretching and good tissue adhesion for hydrogel dressing in real use.

### 2.3 Antibacterial Property

Bacteria tend to adhere to traditional adhesive hydrogels and lead to wound infections.^[^ ^5^ ^]^ Advantages of quaternary ammonium salt antibacterial agents lie in low bactericidal concentration, low toxicity, low irritation, broad‐spectrum, and long‐lasting effects. The principle of sterilization is mainly from the positive charges, which could adsorb the negatively charged bacteria by electrostatic force, and accumulate on their bacterial walls, thus causing growth inhibition and death of bacteria.^[^ ^4^, ^5^, ^33^ ^]^ In this study, highly positive charged pDADMAC brushes of BCD were introduced to provide BCD/PDA/PAM hydrogels with antibacterial property (Figure 1f).^[^ ^17^, ^34^ ^]^

**Figure 4:**
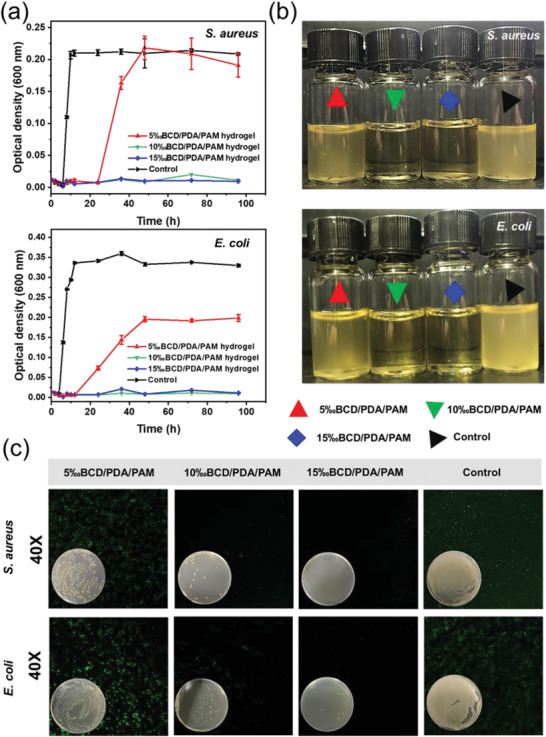
a) Growth curves of *S. aureus* and *E. coli* as a function of culture time in the groups of 5‰BCD/PDA/PAM, 10‰BCD/PDA/PAM, and 15‰BCD/PDA/PAM hydrogels, and the control group (culture solution). The error bars showed standard deviation (*n* = 3). b) Digital photos of the *S. aureus* (10^5^ per CFU, 4 mL) and *E. coli* (10^5^ per CFU, 4 mL) solutions cocultured with the hydrogels after 48 h. c) Live/dead staining of *S. aureus* and *E. coli* after cocultured with the hydrogels for 24 h. The inset digital photos showed the bacterial colonies of *S. aureus* and *E. coli* on agar plates for 24 h.

Contact sterilization experiments were performed by immersing BCD/PDA/PAM hydrogels into *Staphylococcus aureus (S. aureus) and Escherichia coli (E. coli)* culture solutions to assess antibacterial property. The experimental groups were BCD/PDA/PAM hydrogels with different BCD ratios immersed in culture solutions containing bacteria, while the control group was only a culture solution containing bacteria. Optical density (OD_600_) values of bacteria cultured at different times were measured. As shown in **Figure** **4**a, the OD_600_ values of *S. aureus* and *E. coli* in the control group increased significantly at the 8^th^ and 6^th^ hours, respectively, and reached a peak at the 12^th^ hour. In the experimental groups, the OD_600_ values of *S. aureus* and *E. coli* for 10‰BCD/PDA/PAM and 15‰BCD/PDA/PAM hydrogels did not increase within measurement time of 96 h, indicating that the bacteria were completely killed. For 5‰BCD/PDA/PAM hydrogel, the OD_600_ value of *S. aureus* began to increase after 24 h and reached a peak value at the 48^th^ hour, and the OD_600_ value of *E. coli* started to increase after 12 h and also reached a peak value after 48 h. This showed that the higher the BCD ratio, the better the antibacterial performance, and the longer was the antibacterial duration. The degree of turbidity of bacterial culture solutions was related to the number of bacteria.^[^ ^11^ ^]^ It was observed that the culture solutions of 10‰BCD/PDA/PAM and 15‰BCD/PDA/PAM hydrogels were clear after 48 h, while the culture solutions of 5‰BCD/ PDA/PAM hydrogel and the control group were still cloudy after 48 h, which was consistent with the bacterial growth curves (Figure 4b).

A small amount of bacterial solutions was also extracted for live/dead bacteria experiments (Figure 4c) and agar plate experiments (Figure 4c, inset) after 24 h. All bacteria in the group of 15‰BCD/PDA/PAM hydrogel were basically killed. The group of 10‰BCD/PDA/PAM hydrogel was scattered with a small amount of bacteria. The group of 5‰BCD/PDA/PAM hydrogel had a moderate amount of bacteria, while the control group contained a large amount of bacteria. The results clearly indicated when the BCD content exceeded 10‰, BCD/PDA/PAM hydrogels showed a highly effective and long‐lasting antibacterial effect.

### 2.4 Biocompatibility

**Figure 5:**
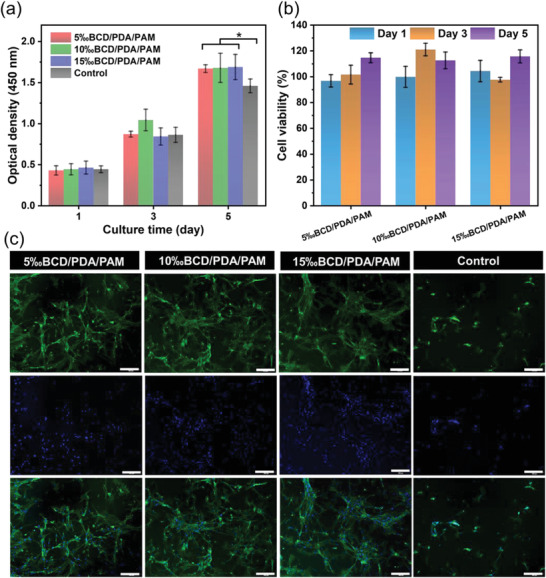
a) CCK‐8 assays of BMSCs and b) cell viability for BCD/PDA/PAM hydrogels after 1, 3, and 5 days of culture. The error bars showed standard deviation (*n* = 3), *p < 0.05 (one‐way ANOVA followed by Bonferroni's multiple comparison test). c) Fluorescence microscope of BMSCs on the groups of BCD/PDA/PAM hydrogels and the control group (culture solution) after 3 days of culture. Scale bar: 200 µm.

Good biocompatibility is another prerequisite for hydrogels used in wound healing.^[^ ^5^, ^30^ ^]^ Presence of PDA and positively charged BCD could enhance the cell adhesion, colonization, and proliferation.^[^ ^26^, ^28^ ^]^ To examine their cytocompatibility, BCD/PDA/PAM hydrogels with different BCD ratios were exposed to mouse bone marrow‐derived mesenchymal stem cells (BMSCs), and presence of viable cells was assessed by cell counting kit‐8 reagent (CCK‐8) assay.^[^ ^12^, ^22^ ^]^ BMSCs were planted on hydrogels and co‐cultured for 5 days. OD_450_ values showed that the proliferation activity of BMSCs on the experimental groups of BCD/PDA/PAM hydrogels and the control group increased gradually with increasing the cultural time (**Figure** **5**a). More importantly, on day 5, OD_450_ values of BCD/PDA/PAM hydrogels were higher than the control group (*p* < 0.05) (Figure 5a). The cell viability of all samples reached almost above 90% within the testing time, confirming the nontoxic nature of BCD/PDA/PAM hydrogels. On the other hand, the cell viability of all BCD/PDA/PAM hydrogels was above 100% on day 5 (Figure 5b). The results about OD_450_ values and cell activity confirmed that BCD/PDA/PAM hydrogels not only had good cytocompatibility, but also could further promote cell proliferation. This might be because BCD/PDA/PAM hydrogels had cell‐adhesive PDA, electrostatically attractive BCD and biomimetic porous structure, and could facilitate attachment, spreading and growth of cells. To visualize the cell viability more intuitively, the immunofluorescence staining of cultured BMSCs on the hydrogels was used by DAPI and Actin‐Tracker Green.^[^ ^11^ ^]^ BMSCs displayed normal cytoskeleton (green) and cell nuclei (blue) morphologies on all the hydrogels. For the hydrogels, the cells were distributed in a spindle‐like shape and formed a higher density of homogeneous cell layer, demonstrating better cell attachment, spreading and retention performances than the control group (Figure 5c). This result was also supported by the live/dead cell assay (Figure S13, Supporting Information). In all groups of BCD/PDA/PAM hydrogels, BMSCs, with a spindle‐like morphology, were green and almost no dead cells were seen after incubation on days 1, 3, and 5. In the control group, the majority of BMSCs were also green, although several dead cells were observed. Therefore, the good biocompatibility made BCD/PDA/PAM hydrogels safe candidate materials for hydrogel dressings.

### 2.5 In Vivo Wound Healing

**Figure 6:**
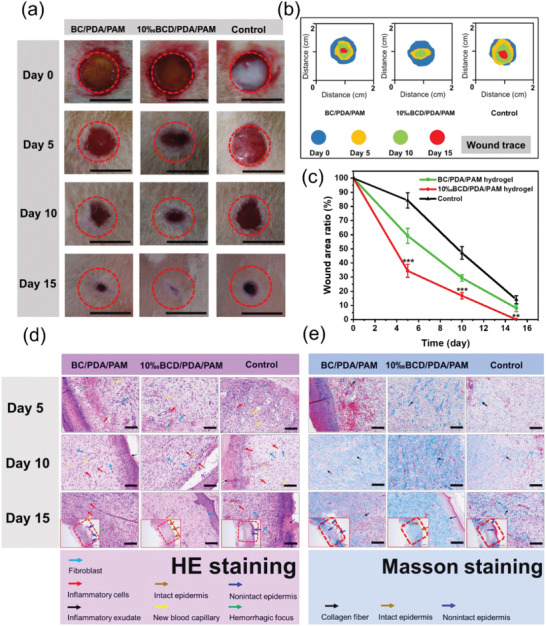
a) Representative digital photos of wounds in the groups of BC/PDA/PAM and 10‰BCD/PDA/PAM hydrogels, and the control group without hydrogel dressing treatment from day 0 to day 15. Scale bar: 10 mm. b) Wound traces at different periods. c) Evolution of wound area ratio at different days for each group. The error bars showed standard deviation (*n* = 3), **p < 0.01 or ***p < 0.001 (one‐way ANOVA followed by Bonferroni's multiple comparison test). d) HE staining and e) Masson staining on day 5, 10, and 15 of the newly regenerated skin tissues for each group. Scale bar: 100 µm.

The ideal wound dressing should have the advantages of promoting healing, anti‐infection, and less irritation.^[^ ^5^, ^11^, ^30^ ^]^ As shown in Figure S14 (Supporting Information), we evaluated the healing‐promoting property of BCD/PDA/PAM hydrogel in a rat's infected wound model and further evaluated the in‐vivo biocompatibility. 10‰BCD/PDA/PAM hydrogel and BC/PDA/PAM hydrogel were chosen as the experimental groups, while the control group received no hydrogel dressing treatment. Considering the wound in normal rats recovered in 2 weeks,^[^ ^11^, ^22^, ^24^ ^]^ the wound healing was evaluated on day 0, 5, 10, and 15. After hydrogel implantation, wounds covered by hydrogels were observed to heal faster than wound in the control group, and redness and swelling of new tissues became less (**Figure** **6**a). This was mainly because of good biocompatibility and cell adhesion derived from catechol groups.^[^ ^22^, ^28^, ^31^ ^]^ Moreover, the multiple network structure constructed with addition of BCD or BC was also beneficial to cell crawling and colonization.^[^ ^35^ ^]^ In order to visualize the change of wound area during wound healing, wound trace figures were drawn by ImageJ and PowerPoint softwares (Figure 6b). The wound area ratio, which was defined as the ratio of wound healing area to initial defect area, was used to quantitatively evaluate the wound healing rate of different hydrogel‐treated wound groups (Figure 6c; Table S2, Supporting Information).^[^ ^11^, ^22^, ^24^ ^]^ The wound healing of experimental groups was significantly better than that of the control group. For example, on day 5, wound treated by 10‰BCD/PDA/PAM hydrogel showed the smallest wound area ratio among all groups. It was worthy to note that 10‰BCD/PDA/PAM hydrogel had ≈15% and 50% advantages compared with BC/PDA/PAM hydrogel and the control group (*p* < 0.001), respectively, indicating a faster healing ratio of wounds treated by 10‰BCD/PDA/PAM hydrogel. On day 10, 10‰BCD/PDA/PAM hydrogel remained ≈13% (BC/PDA/PAM hydrogel) and 30% (the control group) advantages in wound area ratio (*p* < 0.001). Furthermore, on day 15, wound treated by 10‰BCD/PDA/PAM hydrogel showed a complete closure, but BC/PDA/PAM hydrogel group and the control group still had wound area ratios of ≈8% and 14% (*p* < 0.01), respectively. These results clearly indicated that 10‰BCD/PDA/PAM hydrogel had much better wound healing effect than BC/PDA/PAM hydrogel and the control group. The reason was that the 10‰BCD/PDA/PAM hydrogel had abundant positive charge, which killed the bacteria in time and reduced the inflammatory response.^[^ ^4^, ^5^, ^17^, ^33^ ^]^ In addition, under the continuous stimulation of positive charges, it could promote crawling and proliferation of fibroblastic cells.^[^ ^28a,c^ ^]^

### 2.6 Histological Analysis

Infected wound healing is a complex process that involves infiltration of inflammatory cells, accumulation of new capillaries, crawling of fibroblasts and deposition of collagen.^[^ ^5^, ^11^, ^22^, ^24^ ^]^ Histological analysis was used to assess the quality of the regenerated epidermis in defects for the experimental groups of 10‰BCD/PDA/PAM and BC/PDA/PAM hydrogels and the control group without hydrogel dressing treatment on days 5, 10, and 15 (Figure 6d). Inflammatory reaction and cell proliferation were estimated from hematoxylin–eosin (HE) staining. On day 5 of HE staining, inflammatory response was observed in all three groups with inflammatory exudation, new capillary formation, and fibroblast proliferation. The inflammatory response of the control group was the most serious; its connective tissue was loose, and local hemorrhagic focus could be seen. On day 10, the control group still showed a severe inflammatory response, showing a large number of inflammatory cells and new capillary. In the group of BC/PDA/PAM hydrogel, inflammation subsided and inflammatory cells decreased. In sharp contrast, the group of 10‰BCD/PDA/PAM hydrogel had the lightest inflammatory response. On the other hand, although neonatal epidermis could be observed in all three groups, the neonatal epidermis for 10‰BCD/PDA/PAM hydrogel was thicker and had a longer migration distance. Moreover, for 10‰BCD/PDA/PAM hydrogel, the boundary between the neonatal epidermis and the surrounding normal epidermis was not obvious, indicating that its healing effect was better. On day 15, the complete epidermal healing was achieved for 10‰BCD/PDA/PAM hydrogel; a large number of tightly‐connected connective tissues including fibroblasts were observed under the skin. On the contrary, in the control group, a large number of inflammatory cells still existed on day 15, demonstrating that the epidermis was not fully healed. In the group of BC/PDA/PAM hydrogel, presence of moderate inflammatory cells was observed on day 15, indicating the healing was insufficient, but its epidermal healing speed was faster than that of the control group.

Masson staining was performed to assess the formation and deposition of collagen.^[^ ^22^, ^24^ ^]^ On day 5, the deposition of collagen in the experimental and control groups distributed sparsely. On day 10 and 15, the group of 10‰BCD/PDA/PAM hydrogel showed denser granulation tissue deposition and better collagen bundles, as indicated by blue staining in full thickness dermal wounds (Figure 6e). In contrast, both the group of BC/PDA/PAM hydrogel and the control group had poorly‐developed shattered collagen bundles and small blue dyeing areas.

The above results showed that BCD/PDA/PAM hydrogels can play a key role in the healing of infected wound. Adhesion and positive charge enrichment of the hydrogels could promote the crawling, adhesion and reproduction of fibroblasts. The hydrogels also facilitated the production and deposition of collagen. On the other hand, the hydrogels had an antibacterial ability to kill the adhesive bacteria, and thus greatly reduced the inflammatory response and accelerated the healing of wounds.

## 3 Conclusions

We have successfully fabricated a kind of multifunctional hydrogel dressings with stretchable, adhesive, and antibacterial properties for wound healing. The synthetic steps were optimized to operate at room temperature and avoid use of weak alkaline buffering solution. The mechanical property, antibacterial ability and biocompatibility of BCD/PDA/PAM hydrogels were greatly enhanced because of the introduction of BCD. The well‐organized hydrogel systems were gifted with good stretchability, toughness and adhesion. In vitro antibacterial experiments showed that BCD/PDA/PAM hydrogels had high‐efficiency and long‐lasting antibacterial property. In vitro cytotoxicity indicated that BCD/PDA/PAM hydrogels were nontoxic and promoted cell growth and proliferation. In vivo wound healing on rats for 15 days treated by 10‰BCD/PDA/PAM hydrogels showed faster tissue regeneration. Collagen deposition was also enhanced with almost no scar formation at the end of 15 days. In conclusion, our BCD/PDA/PAM hydrogels showed high potential in novel wound dressing, especially for the wounds from dynamic active areas, semi‐contaminated incisions, infected surgical wounds, and other wounds that require frequent replacement of dressings.

## 4 Experimental Section

##### Preparation of BC‐Br

The water in BC water‐dispersion was fully replaced by dimethylformamide (DMF) by centrifugation. BC solution (520 mg in 60 mL DMF) was purged with nitrogen (N_2_) for 30 min, followed by addition of 3.2 mL triethylamine. The mixture was stirred in an ice bath, and 4 mL 2‐bromisobutyryl bromide was added in drops within 30 min. The mixture was further kept at 0 °C for 15 min and then stirred at room temperature for 24 h. The targeted BC‐Br was obtained by washing thoroughly with ethanol and deionized (DI) water to remove residual reactants. The final BC‐Br was stored in a 4 °C refrigerator for later use.

##### Preparation of BCD

According to the literature,^[^ ^17^, ^36^ ^]^ BC‐Br (150 mg) and DADMAC (10 mL) were dissolved in water–methanol mixtures (50:50 v/v, 40 mL) in a Schlenk flask. The solution was purged with N_2_ for 30 min and then CuBr (20.2 mg) and *N,N,N,N,N*‐pentamethyldiethylenetriamine (62 µL) were added immediately. The mixture was still stirred with protection of N_2_ for another 10 min and the reaction was carried out at 60 °C for 24 h with stirring. The final BCD was obtained by washing thoroughly with DI water and ethanol, and then stored in a 4 °C refrigerator for later use.

##### Preparation of Hydrogels

The BCD/PDA/PAM hydrogels were prepared via a two‐step process: 1) BCD was firstly dispersed in the aqueous dopamine solution. Then, APS was added into the solution to initiate prepolymerization of dopamine. The mixture was stirred at room temperature for 25 min as the BCD/PDA solution turns brown. 2) Acrylamide, *N,N*‐methylene bisacrylamide and tetramethylethylenediamine were added to BCD/PDA solution under stirring for 10 min. The final mixture was poured into a mold and kept at 60 °C for 3 h to form hydrogel. Finally, the obtained hydrogel was soaked in DI water and 75% ethanol to remove the excess monomers and dried at room temperature for later use. The weight percentages of all chemicals for various BCD/PDA/PAM hydrogels were listed in Table S1 (Supporting Information).

##### Characterization

FT‐IR spectra were obtained by an FT‐IR spectrometer (TENSOR 27, BRUKER, Germany). Zeta potential of hydrogels was measured by using a Zetasizer Nano‐ZS PN3702 system (Malvern Instruments, Worcestershire, England). Surface morphology, internal structure, and element mapping of hydrogels were analyzed by using a field emission scanning electron microscope (FE‐SEM, Hitachi S‐4800) after lyophilization. XPS (Thermo‐VG Scientific ESCALAB 250Xi) with a standard Al Ka X‐ray source (1486.8 eV) was used to analyze the chemical structure. Self‐healing test was done by using a simple cut‐link model. Compressive, tensile, and adhesive tests were performed on a universal mechanical testing machine (WD‐5A, Guangzhou Experimental Instrument Factory, China). The adhesion performance of BCD/PDA/PAM hydrogels were proven by covering on human skin. The cell compatibility of the hydrogels was confirmed before the experiments. These experiments were carried out with the full, informed consent from human subjects.

##### Antibacterial Property

To investigate the antibacterial activity of BCD/PDA/PAM hydrogels, *S. aureus* and *E. coli* were used for the tests. Bacterial growth curve, colonies forming units (CFU) test and live/dead bacteria assay were evaluated. Details were provided in the Supporting Information.

##### In Vitro Cytotoxicity

The BMSCs (SCSP‐405) were used to evaluate the cytotoxicity and cell attachment of BCD/PDA/PAM hydrogels. Details were provided in the Supporting Information.

##### In Vivo Wound Healing

The BC/PDA/PAM and 10‰BCD/PDA/PAM hydrogels were implanted into full‐thickness wounds of a rat model to evaluate their wound healing performances. Details were provided in the Supporting Information.

##### Histological Analysis

On 5^th^, 10^th^, and 15^th^ day postsurgery, the wounds with surrounding tissue were collected carefully, fixed in 10% paraformaldehyde solution, and embedded in paraffin for routine histological processing. According to the standard protocols, tissues with 5 mm thickness were prepared. HE staining was used to assess the morphology and tissue regeneration. Masson staining was used to assess collagen deposition.

##### Statistical Analysis

Statistical analysis was performed using SPSS software (version 22.0 for Windows; SPSS, Chicago, IL, USA). Data was expressed as mean ± standard deviation (SD). Statistical differences were determined using one‐way analysis of variance (ANOVA) followed by a Bonferroni post hoc test for multiple comparisons. The levels of significance were labeled with *p < 0.05, **p < 0.01, and ***p < 0.001.

## Conflict of Interest

The authors declare no conflict of interest.

## Supporting information

Supporting Information
