# Supplementary material for: Highly Stretchable, Adhesive, Biocompatible, and Antibacterial Hydrogel Dressings for Wound Healing
Source: Adv Sci (Weinh). 2021 Mar 5;8(8):2003627. doi: 10.1002/advs.202003627 (PMC8061386; doi:10.1002/advs.202003627)
Supplement: Supplementary file 1 — Supporting Information [file ADVS-8-2003627-s001.pdf]

© 2021 Wiley-VCH GmbH

ADVANCED SCIENCE
Open Access

Supporting Information

for *Adv. Sci.*, DOI: 10.1002/advs.202003627

Highly stretchable, adhesive, biocompatible, and antibacterial hydrogel dressings for wound healing

Zifeng Yang, Rongkang Huang, Bingna Zheng, Wentai Guo, Chuangkun Li, Wenyi He, Yingqi Wei, Yang Du, Huaiming Wang, Dingcai Wu,* Hui Wang**

Supporting Information

Highly stretchable, adhesive, biocompatible, and antibacterial hydrogel dressings for wound healing

Zifeng Yang, Rongkang Huang, Bingna Zheng, Wentai Guo, Chuangkun Li, Wenyi He, Yingqi Wei, Yang Du, Huaiming Wang, Dingcai Wu,* Hui Wang**

Z. Yang, Dr. R. Huang, W. Guo, C. Li, Y. Wei, Dr. Y. Du, Dr. H. Wang, Prof. H. Wang

Department of Colorectal Surgery

The Sixth Affiliated Hospital

Sun Yat-sen University

Guangdong Institute of Gastroenterology

Guangdong Provincial Key Laboratory of Colorectal and Pelvic Floor Diseases

Guangzhou 510655, P. R. China

E-mail: wang89@mail.sysu.edu.cn

Dr. B. Zheng, W. He, Prof. D. Wu

PCFM Lab and GDHPRC Lab

School of Chemistry

Sun Yat-sen University

Guangzhou 510275, P. R. China

E-mail: zhengbn3@mail.sysu.edu.cn

E-mail: wudc@mail.sysu.edu.cn

Experiment details

1. Materials and animals

Bacterial cellulose water-dispersion (BC, 0.65wt%) was purchased from Qihong technology Co. Ltd (Guilin, China). Triethylamine (TEA, 99%), 2-bromisobutyryl bromide (BiBB, 98%), diallyl dimethyl ammonium chloride (DADMAC, 60% in water), *N,N,N,N,N*-pentamethyldiethylenetriamine (PMDETA, 99%), copper (I) bromide (CuBr, >99%), dopamine hydrochloride (DA), acrylamide (AM), *N,N*-methylene bisacrylamide (BIS), tetramethylethylenediamine (TMEDA), ammonium persulfate (APS), dimethylformamide (DMF, 99.8%), methanol (99.8%), and ethanol (99.8%) were purchased from Aladdin Company (Shanghai, China). Phosphate buffer saline (PBS), Staphylococcus aureus (*S. aureus*, ATCC 6538) and Escherichia coli (*E. coli*, ATCC 25922) were obtained from Guangdong Microbial Culture Collection Center. The Cell counting kit-8 (CCK-8), live/dead cell viability kit (SYTO9 dye and PI dye) was bought from Sigma-Aldrich company (Shanghai, China). Calcein AM/PI Double Staining Kit was bought from Dalian Meilun Biotech Co., Ltd (Dalian, China). Mouse bone marrow-derived mesenchymal stem cells (BMSCs, SCSP-405) were purchased from the Cell Bank of the Chinese Academy of Sciences. Deionized (DI) water was used in the experiment. All other chemicals were purchased from Sigma-Aldrich (Shanghai, China). All solvents and chemicals were purchased from commercial sources and used as received, unless otherwise noted. The male Sprague Dawley (SD) rats (12-week-old, 200-240 g) were purchased from Laboratory Animal Center of Sun Yat-sen University (Guangzhou, China). All rats were fed and tested in

accordance with laboratory rules and guidelines. All rat experiments were approved by the Animal Ethics Committee of South China Agricultural University. Histological analysis was tested by Wuhan Servicebio Technology Co.,Ltd (Wuhan, China).

2. Zeta potential measurements

Before analyzing the zeta potential of hydrogels, the lyophilized hydrogels were milled into powder form.[S1] The hydrogel powders were dispersed in water for 10 minutes, and filtered through filter screen to remove large size hydrogel. Finally, the zeta potential of filtrate was measured at 25 °C by using the Zetasizer Nano-ZS PN3702 system (Malvern Instruments, Worcestershire, England).

3. Mechanical properties testing

Self-healing tests

The hydrogels were cut in half along the middle with a clean blade, and then its cut surfaces were rejoined. The self-healing behaviors of the hydrogels were confirmed through tensile performance after 30 minutes and 2 hours.

Compressive tests

Compressive tests were performed on a universal mechanical testing machine (WD-5A, Guangzhou Experimental Instrument Factory, China) at the compressing speed of 5 mm min^{-1}. The tested 10‰BCD/PDA/PAM hydrogel was cylindrical of 12 mm in height and 16 mm in diameter. Compression resilience of the hydrogel was characterized by 5 cyclic loading-unloading compressive tests to a compressive strain of 60%. Every subsequent cycle was conducted after complete recovery of the hydrogel. Compressive strength of the hydrogel was determined by measuring the ratio of the maximum force

required to crush the hydrogel to the contact area.

Tensile tests

BCD/PDA/PAM hydrogels with different BCD contents were molded into dumbbell specimens (12 mm in length, 5 mm in width, and 1 mm in thickness) for tensile test. Tensile property measurements of hydrogels were performed using a universal mechanical testing machine (WD-5A, Guangzhou Experimental Instrument Factory, China) with a 100 N load cell and an extension speed of 15 mm min^{-1}. The cyclic tensile test of 10‰BCD/PDA/PAM hydrogel was characterized by 4 cyclic loading-unloading tensile tests to a tensile strain of 400%.

Adhesive tests

The adhesion strengths of BCD/PDA/PAM hydrogels were evaluated by lap shear tests using the universal mechanical testing machine (WD-5A, Guangzhou Experimental Instrument Factory, China) equipped with a 100 N load cell and a 2 mm min^{-1} loading rate.[S2] Porcine skin which was chosen to mimic the adhesion on human tissue was cut into 20 mm×10 mm and glued to the glass. The tested hydrogels were cut to 10 mm× 10 mm and sandwiched between two pieces of porcine skin for lap shear tests. The adhesion tests were immediately conducted once the hydrogels were quickly attached on the porcine skin. The adhesion strengths were calculated by the maximum load divided by the initial bonded area. Cyclic adhesion tests of BCD/PDA/PAM hydrogels were evaluated. The tests were repeated with three parallel specimens.

4. Antibacterial property of BCD/PDA/PAM hydrogels

Bacterial growth curves

To investigate the antibacterial activity of BCD/PDA/PAM hydrogels, *S. aureus* and *E. coli* were used for the tests.[S3] All the hydrogels were taken under UV exposure for 24 hours before bacterial culture. The original bacterium fluid of *S. aureus* and *E. coli* was inoculated in Luria-Bertani (LB) growth medium for 24 hours at 37 °C with constant shaking. The typical colony was taken out by an inoculation ring to 50 mL of nutrient broth at 37 °C for 12 hours. The concentration of bacteria was 10^7 colonies forming units (CFU)/mL. The resulting *S. aureus* and *E. coli* suspensions were stored in a sterile medical bottle. The solution was further diluted 100 times to 10^5 CFU/mL. *S. aureus* or *E. coli* suspensions (4 mL) were added to the small glass bottles containing BCD/PDA/PAM hydrogels (size: 15 mm ×15 mm×1 mm). In this assay, a bacterial solution without any treatment was used as the control group. Each sample was repeated for three times. The bottles were incubated at 37 °C for 24-96 hours with 180 rpm. During the incubation, the optical density (OD_{600}) value of the above bacterial solutions was measured at different times. Meanwhile, the turbidity change of bacterial liquids at different times was observed.

CFU test and live/dead bacteria assay

Briefly, after incubation at 37 °C for 24 hours with 180 rpm, 15 μL of the bacterial solution was uniformly spread on an agar plate. After incubating on agar plate at 37 °C for another 24 hours, the bacterial colony forming units were photographed. Meanwhile, the hydrogels were taken out and washed with PBS for 2-3 times. Subsequently, the hydrogels were soaked in the solution containing 1.5 μL SYTO9 dye, 1.5 μL PI dye and 997 uL DI water, and continued to incubate at room temperature for 15 min in the dark.

Finally, the hydrogels were washed three times with PBS and then visualized using a fluorescence microscope (Olympus IX73, Japan).

5. In vitro cytotoxicity

The mouse bone marrow-derived mesenchymal stem cells (BMSCs, SCSP-405) were resuspended in a complete culture medium consisting of mesenchymal stem cell medium (MSCM) with 5% w/v fetal bovine serum (FBS), 0.5 mL mesenchymal stem cell growth additive and 0.5 mL penicillin/streptomycin solution, and then were cultured in a carbon dioxide cell incubator (37 °C, 5% CO_2). The complete medium was replaced every two days to achieve the purpose of cell proliferation. Cellular cytotoxicity and proliferation were measured using cell counting kit-8 reagent (CCK-8, Domino, Japan). Specifically, the hydrogels were cut to an appropriate size and placed in the bottom of a 96-well plate. After soaking in a mixture of absolute ethanol and PBS (v/v = 75%) for 12 hours for sterilization, the hydrogels were washed with PBS for 5 times to remove ethanol. Subsequently, BMSCs were seeded into hydrogels as a cell density of 2×10^4 /well, placed in carbon dioxide cell incubator (37 °C, 5% CO_2) and continued to culture until the cells returned to the normal adherent state. 10% CCK-8 reagent was added to the corresponding wells at different time points (Day 1, 3, and 5), and the incubation was continued for 2 hours. Cells seeded into culture without hydrogel served as the control group. The obtained supernatants were transferred to another 96-well plate. Then, the optical density at 450 nm (OD_{450}) was measured by a microplate reader, and the cell viability was calculated.

Effect of BCD/PDA/PAM hydrogels on the cell viability was observed by

fluorescence staining. Briefly, BMSCs were seeded onto the hydrogels and cultured for 3 days. Subsequently, all cell substrates were fixed on ice with 4% paraformaldehyde for 15 minutes, followed by washing three times with PBS. Then permeabilization of the cells was done with 0.1% Triton-PBS for 10 minutes, followed by washing three times with PBS. After that, DAPI (Beyotime, China) was used to visualize the cell nucleus, while Actin-Tracker Green (Beyotime, China) was utilized to show cytoskeleton. After staining in the dark, the fluorescence images were taken using a fluorescence microscope (Olympus IX73, Japan).

Live/dead cell assay of BMSCs encapsulated in BCD/PDA/PAM hydrogels was conducted via a Calcein AM/PI Double Staining Kit according to instructions from the manufacturer. The experiments were carried out on day 1, 3 and 5 post-seeding. The hydrogels were visualized using a fluorescence microscope (Olympus IX73, Japan).

For the cell attachment experiment, PAM, PDA/PAM, 10‰BCD/PAM and 10‰BCD/PDA/PAM hydrogels of each group (n = 3) were placed into 24-well plates. The cells were seeded at a density of 1×10^4 cells/cm^2. Briefly, 500 μL of cell suspension was gently added to the hydrogels along the edge of culture plate. After 24-hour culture, the above cells were fixed with 2.5% glutaraldehyde aqueous solution for 15 minutes, and washed with PBS three times to remove residual glutaraldehyde. Subsequently, the cell morphology and attachment were observed by a fluorescence microscope (Olympus IX73, Japan).

6. In vivo wound healing

Briefly, 6 male Sprague Dawley (SD) rats weighing 200-240 g were used. After being

anesthetized with pentobarbital (2 wt.%, 1.8 mL kg^{-1}), the dorsal area of rats was totally depilated and 3 full-thickness circular wounds (10 mm in diameter) were created on the upper back of each rat by a disposable 10 mm skin biopsy punch.[S4,S5] On each rat, a wound without hydrogel dressing treatment was used as control. After dropping 10 μL of *S. aureus* (10^5 CFU/mL) into the wound, a tailored hydrogel was placed, and a 3M Tegaderm TM (Neuss, Germany) was covered. Then, the healing of the wound was observed and photographed every day. During the observation period, a rat was executed on day 5, 10, and 15, respectively. The wound site from the executed rat was harvested in full layer with scissors in conjunction with surrounding tissues, and then soaked in 10% formalin solution. Paraffin embedding was performed as soon as possible for subsequent histological analysis.

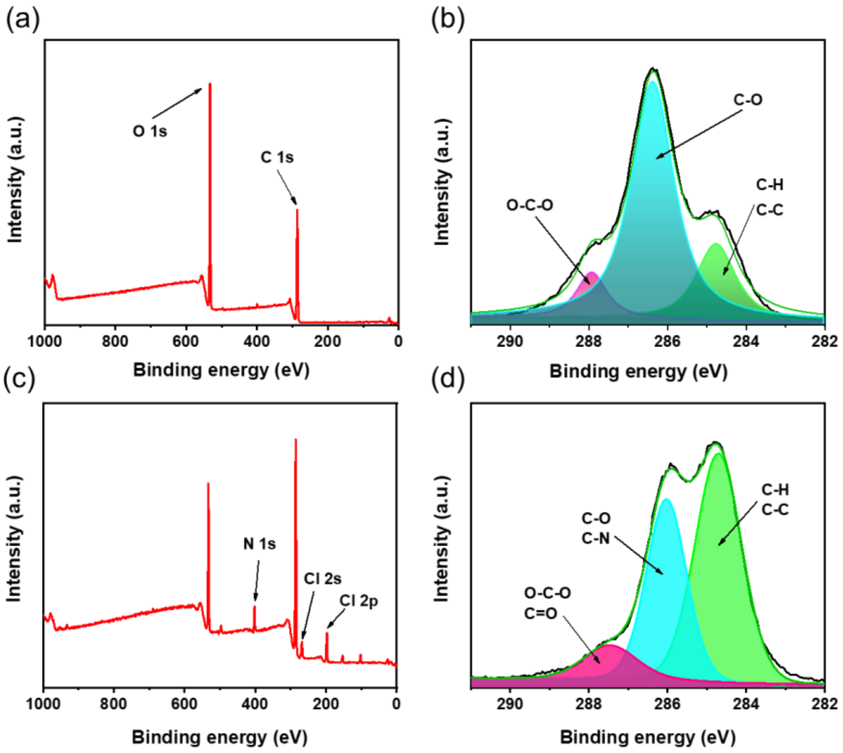

Figure S1. XPS spectra of (a, b) BC and (c, d) BCD.

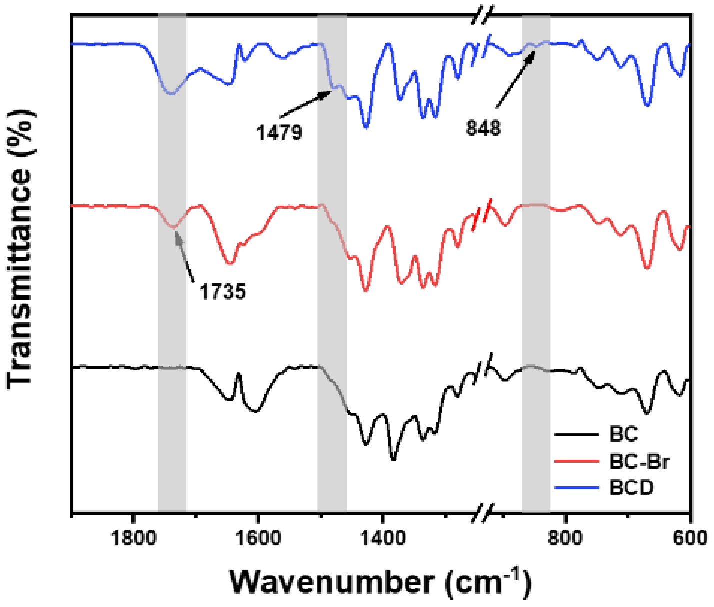

Figure S2. FT-IR spectra of BC, BC-Br and BCD.

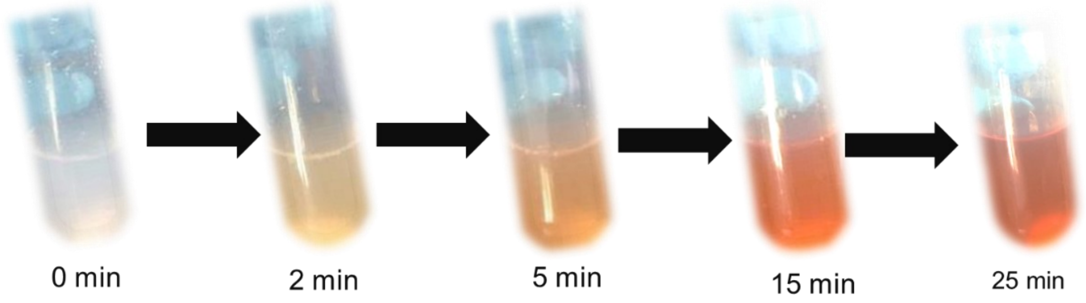

Figure S3. The solution colors at different reaction times in the process of prepolymerization of dopamine.

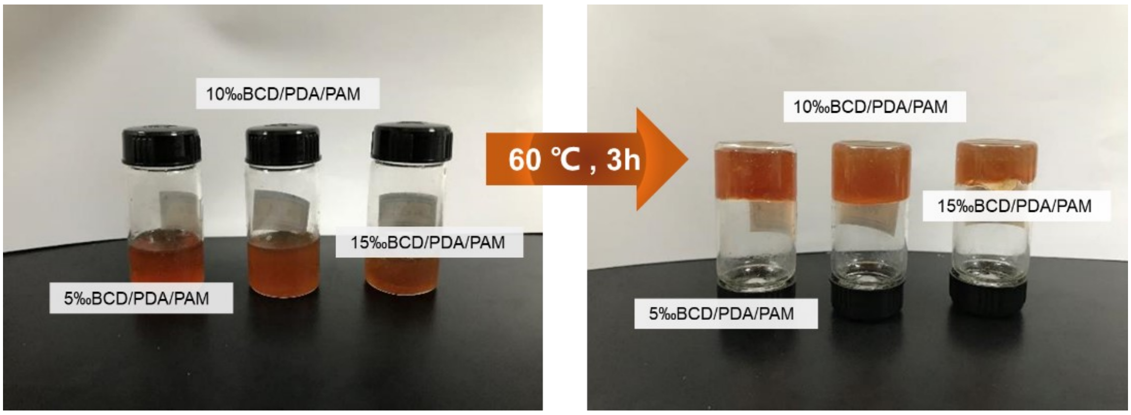

Figure S4. Solid hydrogels formed after 3 hours of reaction at 60°C.

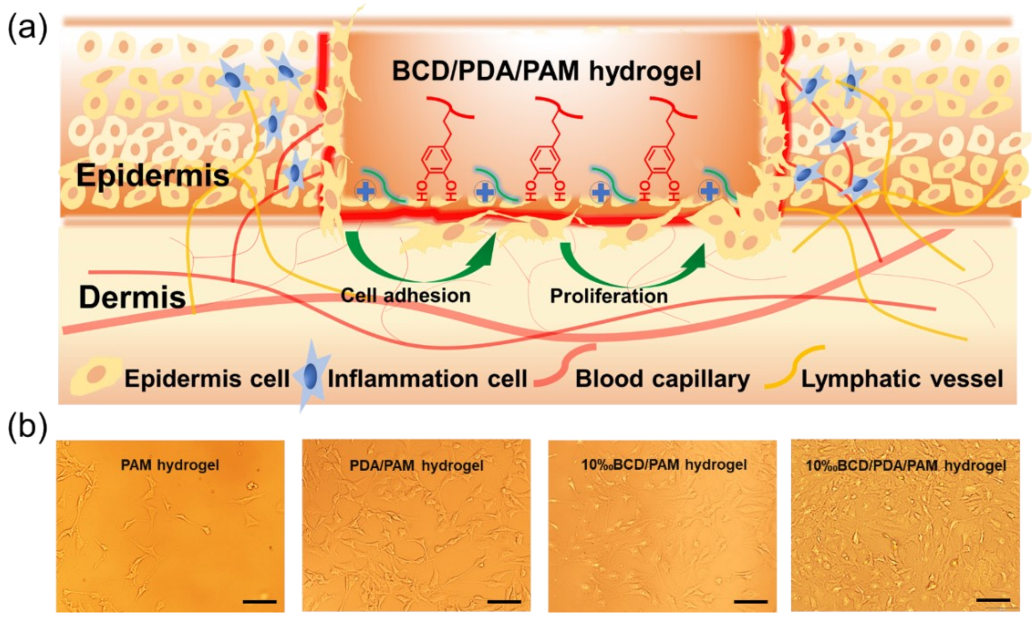

Figure S5. (a) Catechol groups and positive charges promote cell adhesion and proliferation. (b) Micrographs of BMSCs on the surface of PAM, PDA/PAM, 10‰BCD/PAM and 10‰BCD/PDA/PAM hydrogels after 24-hour culture. The more the attached cells, the better was the cell affinity. It was found that more cells were attached on the surface of PDA/PAM, 10‰BCD/PAM and 10‰BCD/PDA/PAM hydrogels compared with the PAM hydrogel after 24-hour culture, verifying the good cell affinity of PDA and BCD components. Scale bar: 100 μm.

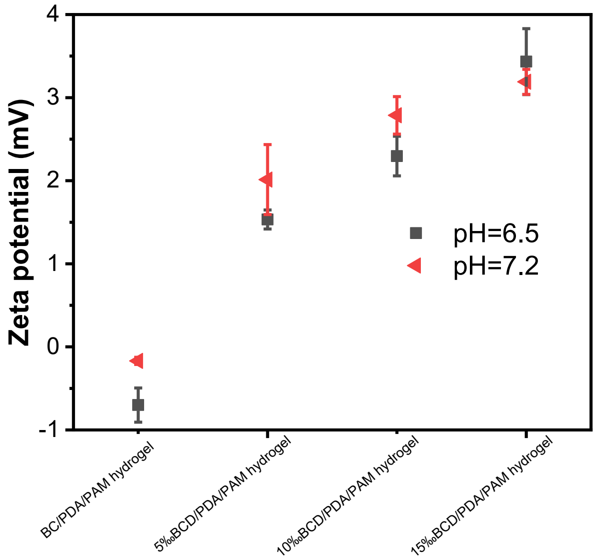

Figure S6. Zeta potential of BC/PDA/PAM and BCD/PDA/PAM hydrogels at pH=6.5 and 7.2.

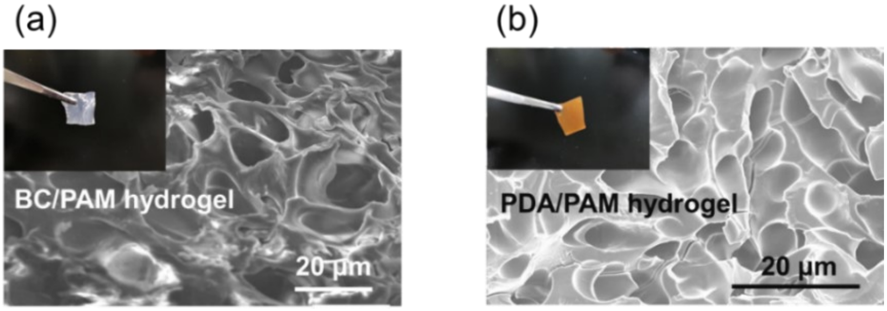

Figure S7. SEM images and digital photos (inset) of (a) BC/PAM and (b) PDA/PAM hydrogels.

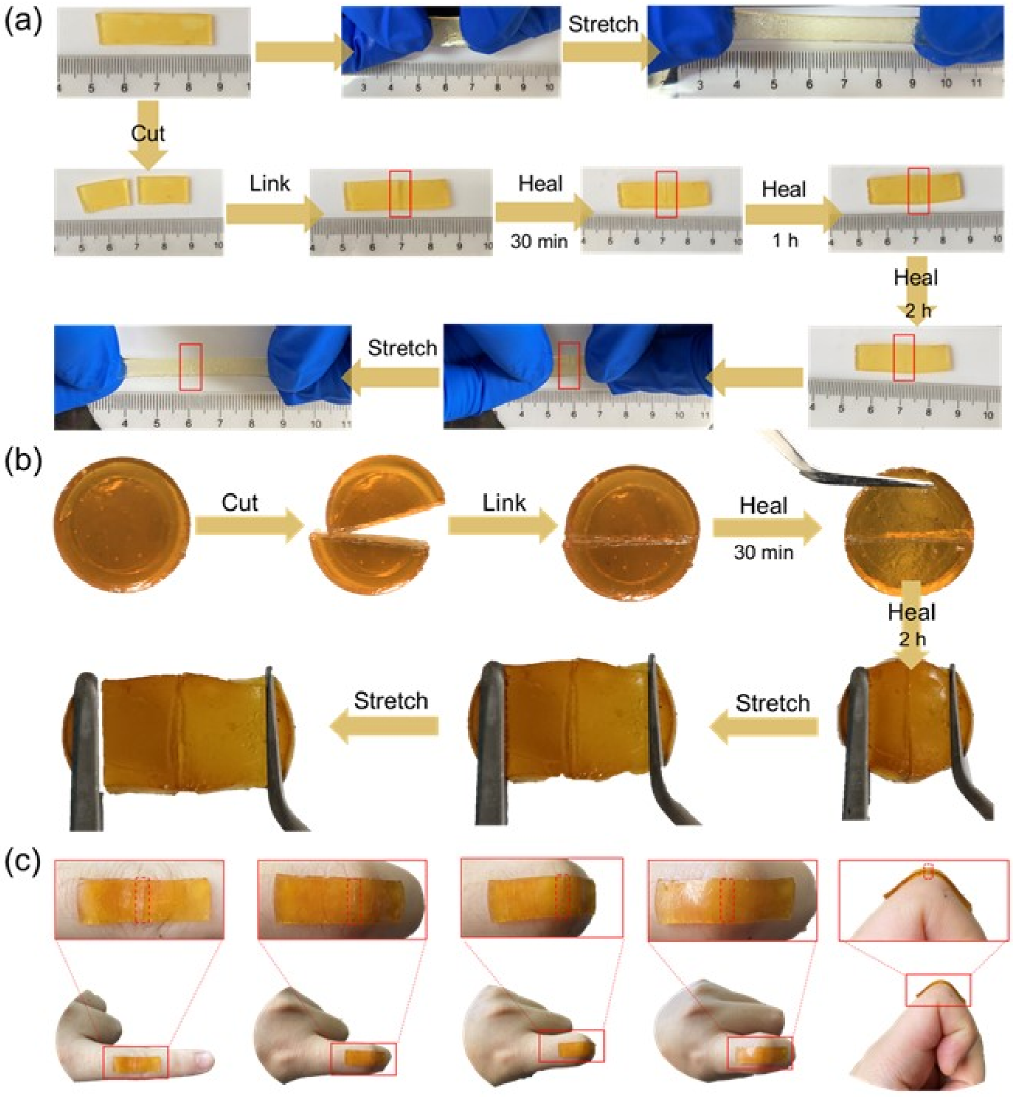

Figure S8. (a, b) Self-healing performance of 10‰BCD/PDA/PAM hydrogel. (c) No re-fracture for the 30-minute self-healed 10‰BCD/PDA/PAM hydrogel attached to the left index finger with the movement of joints.

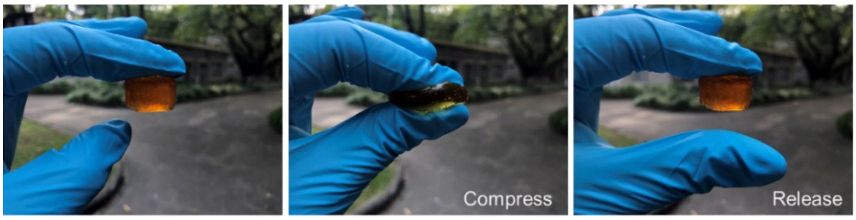

Figure S9. Digital photos of compressive loading-unloading test of 10‰BCD/PDA/PAM hydrogel.

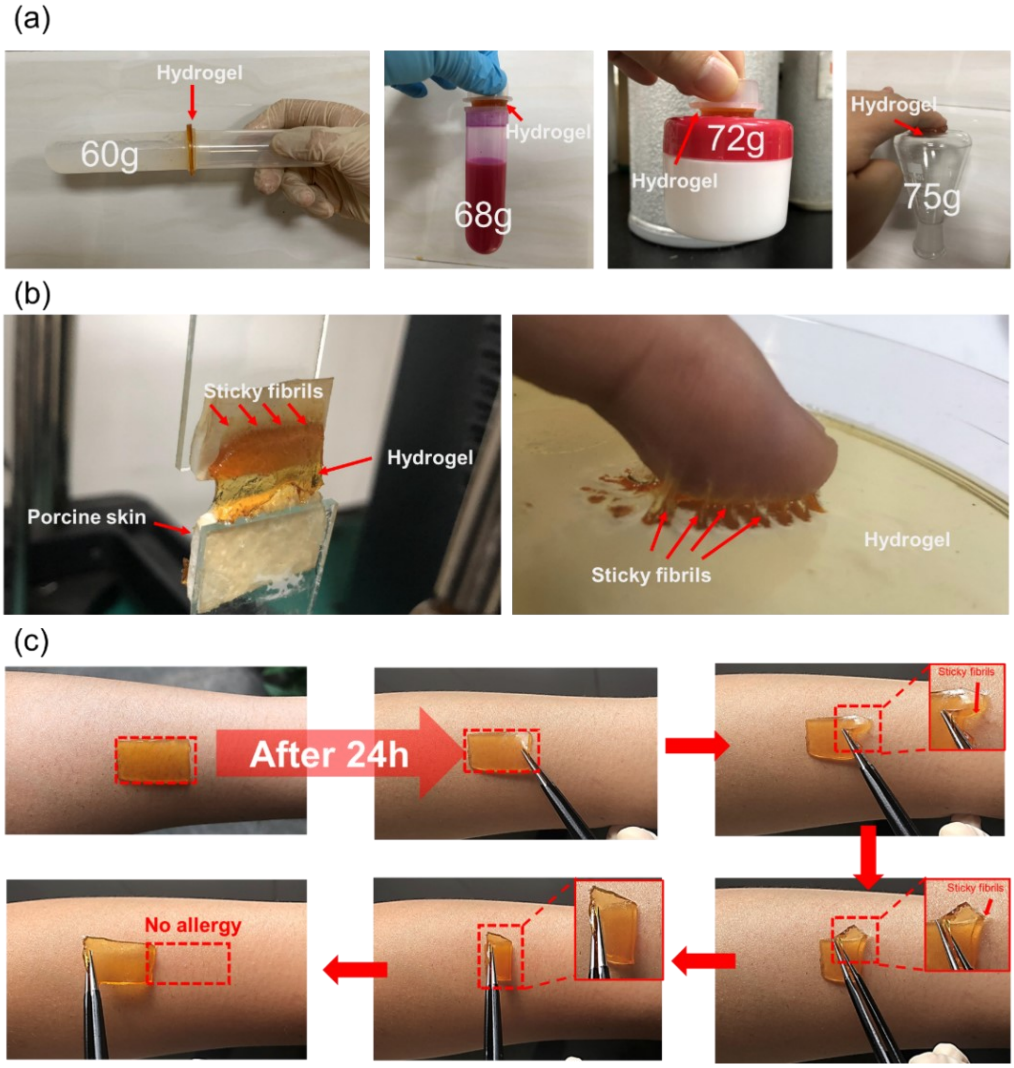

Figure S10. (a) Digital photos of 10‰BCD/PDA/PAM hydrogel adhered to various materials with different bearing weights: plastic with 60, 68 and 72 g, and glass with 75 g. (b) Sticky fibrils could be observed as the hydrogel adhered on the porcine skin and author's finger. (c) The hydrogel was still adhered on human's skin after 24 h and no allergy happened after removing the hydrogel.

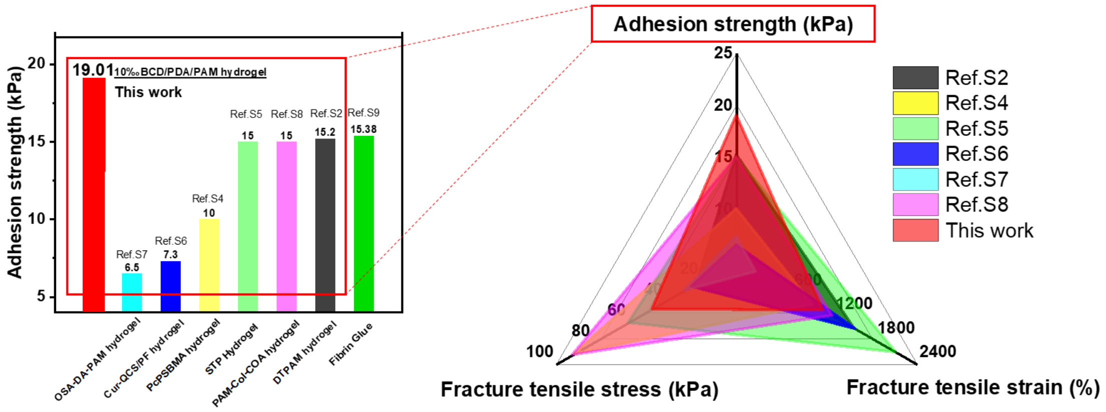

Figure S11. Comparisons of adhesion strength, fracture tensile stress and fracture tensile strain between 10‰BCD/PDA/PAM hydrogel and other reported hydrogel dressings.

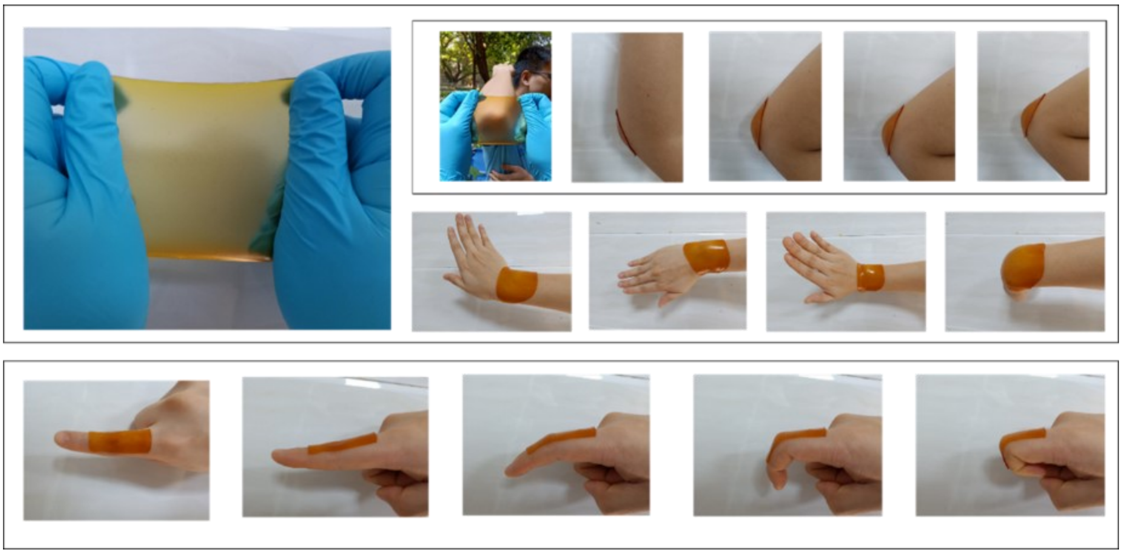

Figure S12. Digital photos of 10‰BCD/PDA/PAM hydrogel adhered on elbow, dorsal side of wrist and finger with dynamic stretching along with the movement of joints.

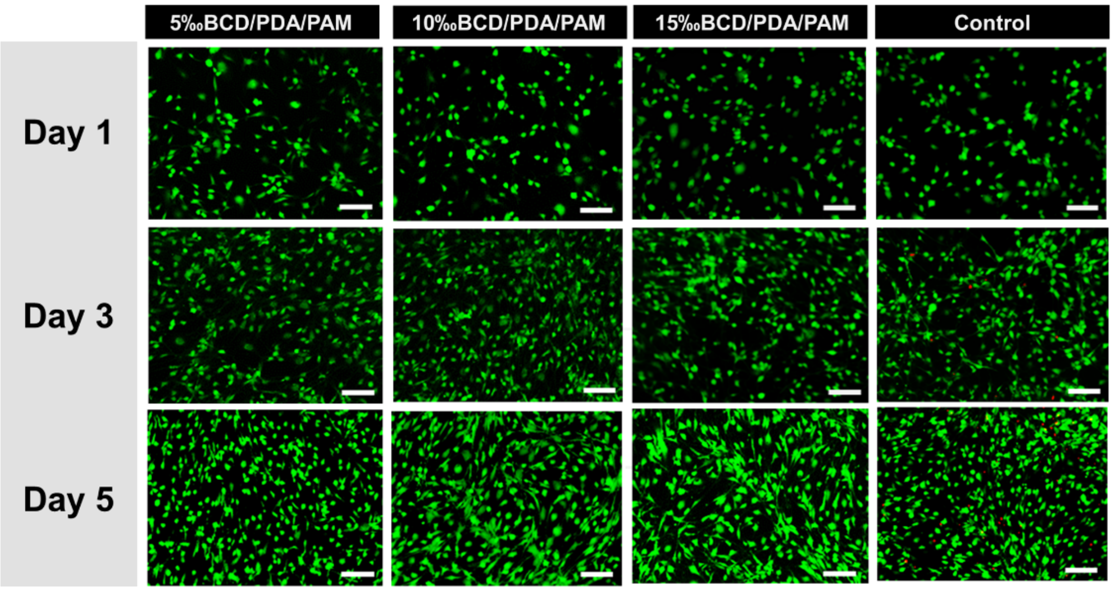

Figure S13. Live/dead cell staining of BMSCs encapsulated in the groups of BCD/PDA/PAM hydrogels and the control group on day 1, 3 and 5. Scale bar: 100 μm.

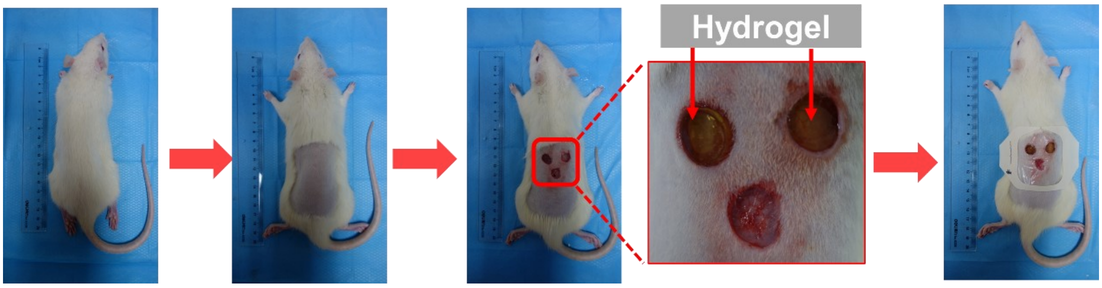

Figure S14. Surgical procedures of BC/PDA/PAM and 10‰BCD/PDA/PAM hydrogels as wound dressings on the wound sites of rat dorsal.

Table S1. Synthesis of BCD/PDA/PAM hydrogels.

Hydrogel	BCD/AM (wt.‰)	DA/AM (wt.%)	AM (g)	APS/AM (wt.%)	BIS/AM (wt.%)	TMEDA (μL)	Water (mL)
**5‰BCD/PDA/PAM**	5	0.6	2.5	8.0	0.6	20	10
**10‰BCD/PDA/PAM**	10	0.6	2.5	8.0	0.6	20	10
**15‰BCD/PDA/PAM**	15	0.6	2.5	8.0	0.6	20	10

Table S2. Wound area ratios on day 5, 10 and 15 for the groups of BC/PDA/PAM and BCD/PDA/PAM hydrogels and the control group.

	**BC/PDA/PAM (%)**	**10‰BCD/PDA/PAM (%)**	**Control (%)**
**Day 5**	59.2 ± 5.2	34.4 ± 4.5	84.3 ± 5.4
**Day 10**	29.4 ± 2.3	16.8 ± 2.2	47.1 ± 4.5
**Day 15**	8.1 ± 2.4	0	14.3 ± 2.8

Reference

[S1] S. Zhang, J. Ermann, M. D. Succi, A. Zhou, M. J. Hamilton, B. Cao, J. R. Korzenik, J. N. Glickman, P. K. Vemula, L. H. Glimcher, G. Traverso, R. Langer, J. M. Karp, *Sci. Transl. Med.* **2015**, 7, 300ra128.

[S2] X. Jing, H. Y. Mi, Y. J. Lin, E. Enriquez, X. F. Peng, L. S. Turng, *ACS Appl. Mater. Inter.* **2018**, *10*, 20897.

[S3] Y. Jia, H. Zhang, S. Yang, Z. Xi, T. Tang, R. Yin, W. Zhang, *Nanomedicine* **2018**, *13*, 2881.

[S4] X. Pei, H. Zhang, Y. Zhou, L. Zhou, J. Fu, *Mater. Horiz.* **2020**, 7, 1872.

[S5] H. Qiao, P. Qi, X. Zhang, L. Wang, Y. Tan, Z. Luan, Y. Xia, Y. Li, K. Sui, *ACS Appl. Mater. Inter.* **2019**, *11*, 7755.

[S6] J. Qu, X. Zhao, Y. Liang, T. Zhang, P. X. Ma, B. Guo, *Biomaterials* **2018**, 183, 185.

[S7] T. Chen, Y. Chen, H. U. Rehman, Z. Chen, Z. Yang, M. Wang, H. Li, H. Liu, *ACS Appl. Mater. Inter.* **2018**, 10, 33523.

[S8] Z. Bai, W. Dan, G. Yu, Y. Wang, Y. Chen, Y. Huang, C. Yang, N. Dan, *RSC Adv.* **2018**, 8, 42123.

[S9] M. Mehdizadeh, H. Weng, D. Gyawali, L. Tang, J. Yang, *Biomaterials* **2012**, 33, 7972.
